# The N terminus-only (*trans*) function of the adhesion G protein-coupled receptor latrophilin-1 controls multiple processes in reproduction of *Caenorhabditis elegans*

**DOI:** 10.1093/g3journal/jkae206

**Published:** 2024-09-07

**Authors:** Daniel Matúš, Willem Berend Post, Victoria Elisabeth Groß, Alexander Bernd Knierim, Christina Katharina Kuhn, Franziska Fiedler, Darian Benno Tietgen, Johanna Lena Schön, Torsten Schöneberg, Simone Prömel

**Affiliations:** Medical Faculty, Rudolf Schönheimer Institute of Biochemistry, Leipzig University, 04103 Leipzig, Germany; Department of Molecular and Cellular Physiology, Stanford University, Stanford, CA 94305, USA; Department of Biology, Institute of Cell Biology, Heinrich Heine University Düsseldorf, 40225 Düsseldorf, Germany; Department of Biology, Institute of Cell Biology, Heinrich Heine University Düsseldorf, 40225 Düsseldorf, Germany; Medical Faculty, Rudolf Schönheimer Institute of Biochemistry, Leipzig University, 04103 Leipzig, Germany; IFB Adiposity Diseases, Leipzig University Medical Center, 04103 Leipzig, Germany; Medical Faculty, Rudolf Schönheimer Institute of Biochemistry, Leipzig University, 04103 Leipzig, Germany; Department of Diagnostics, Fraunhofer Institute for Cell Therapy and Immunology (IZI), 04103 Leipzig, Germany; Medical Faculty, Rudolf Schönheimer Institute of Biochemistry, Leipzig University, 04103 Leipzig, Germany; Department of Biology, Institute of Cell Biology, Heinrich Heine University Düsseldorf, 40225 Düsseldorf, Germany; Medical Faculty, Rudolf Schönheimer Institute of Biochemistry, Leipzig University, 04103 Leipzig, Germany; Department of Dermatology, Venereology and Allergology, Leipzig University Medical Center, Leipzig University, 04103 Leipzig, Germany; Medical Faculty, Rudolf Schönheimer Institute of Biochemistry, Leipzig University, 04103 Leipzig, Germany; School of Medicine, University of Global Health Equity, Kigali 6955, Rwanda; Department of Biology, Institute of Cell Biology, Heinrich Heine University Düsseldorf, 40225 Düsseldorf, Germany

**Keywords:** adhesion GPCR, latrophilin, *trans* function, germ cells, sperm guidance, ovulation, apoptosis

## Abstract

Adhesion G protein-coupled receptors are unique molecules. They are able to transmit classical signals via G protein activation as well as mediate functions solely through their extracellular N termini, completely independently of the seven transmembrane helices domain and the C terminus. This dual mode of action is highly unusual for G protein-coupled receptors and allows for a plethora of possible cellular consequences. However, the physiological implications and molecular details of this N terminus-mediated signaling are poorly understood. Here, we show that several distinct seven transmembrane helices domain-independent/*trans* functions of the adhesion G protein-coupled receptor latrophilin homolog latrophilin-1 in the nematode *Caenorhabditis elegans* together regulate reproduction: sperm guidance, ovulation, and germ cell apoptosis. In these contexts, the receptor elicits its functions in a noncell autonomous manner. The functions might be realized through alternative splicing of the receptor specifically generating N terminus-only variants. Thus, our findings shed light on the versatility of seven transmembrane helices domain-independent/N terminus-only/*trans* functions of adhesion G protein-coupled receptor and discuss possible molecular details.

## Introduction

Cellular communication mediated by G protein-coupled receptors (GPCR) typically involves the transduction of extracellular signals into a cell, primarily realized through intracellular activation of G proteins via the receptor's seven transmembrane helices domain (7TM). This concept has also been confirmed for most members of the class of adhesion GPCR (aGPCR) (Bohnekamp and Schöneberg 2011; Gupte *et al*. 2012). Like other GPCR, these receptors play essential roles in various physiological and pathophysiological processes (summarized in Hamann *et al*. 2015; Rosa *et al*. 2021; Einspahr and Tilley 2022). However, they harbor structural features that distinguish them as a separate class within the GPCR superfamily (summarized in Hamann *et al*. 2015; Liebscher *et al*. 2022; Seufert *et al*. 2023). One of these features is their extraordinarily long extracellular N terminus comprising various domains. This structure enables the receptors to mediate signals and participate in cell–cell or cell–matrix adhesion (summarized in Hamann *et al*. 2015).

Remarkably and in contrast to other GPCR, members of the class of aGPCR are capable of transmitting functions solely via these complex N termini, indicating noncanonical signaling mechanisms completely independent of their 7TM and C terminus (N terminus-only/7TM-independent/*trans* function). This intriguing mode of function is in stark contrast to the canonical 7TM-dependent/*cis* signaling employed by all GPCR, which requires the entire receptor molecule and is limited to transmitting signals into the cell the receptor is expressed on. Conversely, 7TM-independent/*trans* functions have the potential to affect adjacent cells (potentially noncell autonomously) by mediating adhesion or even activation of other surface receptors. This dual mode of action is highly uncommon for GPCR. Our work and that of others suggest that several aGPCR, such as latrophilins/LPHN/ADGRL (Prömel *et al*. 2012; Matus *et al*. 2022), BAI1/ADGRB1 (Tu *et al*. 2018), GPR126/ADGRG6 (Patra *et al*. 2013), and CD97/ADGRE5 (Ward *et al*. 2018), harbor the ability to act in such bidirectional manner, with evidence for more (Okajima *et al*. 2010; Steimel *et al*. 2010; Patat *et al*. 2016). These cases imply that the unique dual mode of function is a common feature of this receptor class, and data from several studies indicate that the sole N terminus independent of the 7TM and C terminus is always sufficient to mediate effects (Prömel *et al*. 2012; Patra *et al*. 2013; Tu *et al*. 2018; Ward *et al*. 2018; Matus *et al*. 2022). However, while evidence for the existence of N terminus-only/7TM-independent/*trans* functions is growing and models of their possible cellular consequences are beginning to take shape, essential questions regarding the molecular framework of this concept remain unanswered. For instance, while it was observed that this mode of function affects cells surrounding the aGPCR-expressing cells, it is not shown whether the receptor is activating signaling cascades in these cells or whether its noncell autonomous effect is mediated, for example, via adhesion. Furthermore, it is unclear whether the whole receptor molecule is intact at all times or whether the N terminus could be released or produced independently of the C-terminal parts to mediate its function. Autocatalytic cleavage at the G protein-coupled receptor proteolysis site (GPS) (Lin *et al*. 2004) yielding an N-terminal fragment and a C-terminal fragment occurs in many aGPCR and might offer a possible mechanism for realizing the N terminus-only/7TM-independent/*trans* function. However, our previous work showed that this cleavage is not absolutely vital for the 7TM-independent receptor mode of latrophilin-1 (LAT-1), a latrophilin homolog in the nematode *Caenorhabditis elegans* (Prömel *et al*. 2012), thus indicating that other mechanisms exist. More attempts to understand the mechanistic details of the N terminus-only/7TM-independent/*trans* aGPCR functions have been made in vitro and ex vivo (Okajima *et al*. 2010; Tu *et al*. 2018; Ward *et al*. 2018). However, these systems are not too well equipped to address the noncell autonomous roles of aGPCR, how they act in diverse multicellular settings, and how they are connected to and integrated with the classical G protein-mediated receptor functions. In vivo studies are more suitable to grasp the integration of the classical G protein-mediated and the 7TM-independent function and their physiological consequences.

Here, we provide evidence that the N terminus-only/7TM-independent/*trans* function of the aGPCR LAT-1 in *C. elegans* is vitally involved in a wide spectrum of physiological processes within the nematode. Previous studies have shown that the receptor controls anterior–posterior cell division plane orientation in the early *C. elegans* embryo (Langenhan *et al*. 2009) via *cis* signaling (Prömel *et al*. 2012) through a Gs protein cascade (Müller *et al*. 2015). Furthermore, an N terminus-only/7TM-independent/*trans* function has been described in neuron morphogenesis (Matus *et al*. 2022) and in reproduction (Prömel *et al*. 2012). In the current study, we show that LAT-1 regulates sperm guidance, ovulation, germline apoptosis, and potentially the quality of germ cells. All these functions together cumulate in contributing to reproduction and brood size control. At first glance, *lat-1* expression in both the affected germ cells and the surrounding somatic cells of the gonad does not necessarily imply a noncell autonomous mechanism. However, we show that sole *lat-1* expression in the cells of the somatic gonad is sufficient for many of its functions in the reproductive system. Additionally, we discuss the possibility of realizing isolated *trans* functions through alternative splicing of the receptor specifically generating N terminus-only variants.

## Materials and methods

### Materials and reagents

All standard chemicals were from Sigma-Aldrich, Thermo Fisher Scientific, or Carl Roth unless stated otherwise. All enzymes were obtained from New England Biolabs.

### *Caenorhabditis elegans* maintenance and strains

*C. elegans* strains were maintained according to standard protocols (Brenner 1974) on *Escherichia coli*OP50 at 22°C unless stated otherwise. Strains carrying *nIs13 [pie-1p::vab-1::GFP + unc-119(+)]; ltIs44 [pie-1p::mCherry::PH(PLC1delta1) + unc-119(+)]* were kept at 25°C.

Wild-type worms were *C. elegans* var. Bristol strain N2 (Brenner 1974). The following alleles were obtained from the *Caenorhabditis* Genetics Center (CGC), which is funded by the NIH Office of Research Infrastructure Programs (P40 OD010440): *lat-1(ok1465)* (generated by the *C. elegans* gene knockout consortium), *qIs153[lag-2p::MYR::GFP; ttx-3p::DsRed] (*Byrd *et al*. 2014*)*, *nIs13[pie-1p::vab-1::GFP; unc-119(+)]*; *ltIs44[pie-1p::mCherry::PH(PLC1delta1)*; *unc-119(+)]* (Cheng *et al*. 2008), *tnIs6[lim-7p::GFP; rol-6(su1006)]* (Hall *et al*. 1999), and *bcIs39[lim-7p::ced-1::GFP; lin-15(+)]* (Zhou *et al*. 2001). The following strains containing extrachromosomal or integrated arrays were generated in this study: *lat-1(ok1465) aprEx192[pTL20; myo-2::mCherry; pBSK]*, *lat-1(ok1465) aprEx193[pJL13; myo-2p::mCherry; pBSK]*, *lat-1(ok1465) aprEx194[pJL14; myo-2::mCherry; pBSK]*, *lat-1(ok1465) aprEx216[pDM3; myo-2::mCherry; pBSK]*. Strains *lat-1(knu846[lat-1 KO/KI mCherry intronic loxP::hygR::loxP])* [NemaMetrix Inc. (Eugene, OR, USA)] and *lat-1(apr1[lat-1(1-581)::GFP^hygR^])* were generated using CRISPR/Cas9 genome editing. The following combinations of transgenes/alleles were obtained using standard genetic techniques (Brenner 1974): *lat-1(knu846 [lat-1 KO/KI mCherry intronic loxP::hygR::loxP])*; *tnIs6[lim-7p::GFP; rol-6(su1006)]*, *lat-1(knu846[lat-1 KO/KI mCherry intronic loxP::hygR::loxP])*; *qIs153[lag-2p::myr::GFP; ttx-3p::DsRed]*, *lat-1(ok1465)*; *nIs13[pie-1p::vab-1::GFP; unc-119(+)]*; *ltIs44[pie-1p::mCherry::PH(PLC1delta1)*; *unc-119(+)]*, *lat-1(ok1465)*; *nIs13[pie-1p::vab-1::GFP; unc-119(+)]*; *ltIs44[pie-1p::mCherry::PH(PLC1delta1)*; *unc-119(+)]; aprEx192[pTL20 myo-2::mCherry pBSK]*, and *bcIs39[lim-7p::ced-1::GFP; lin-15(+)]; lat-1(ok1465).*

### Generation of transgenic *C. elegans* lines

All transgenic strains with stably transmitting extrachromosomal arrays were generated using DNA microinjection. Plasmids were injected at a concentration of 1 ng/μl together with the coinjection marker, a modified pPD118.33 containing *myo-2p::mCherry* (kind gift of Ralf Schnabel (Technical University Braunschweig, Germany) (30 ng/μl), and pBluescript II SK + vector DNA (Stratagene) as stuffer DNA to achieve a final concentration of 120 ng/μl. DNA was injected into the syncytial gonad of hermaphrodites. Transgenic progeny were isolated, and stable lines were selected. Multiple independent transgenic lines were established for each transgene tested.

For the CRISPR/Cas9-mediated genetic modifications, the injection cocktail consisted of *Peft-3::cas9-SV40_NLS::tbb-2 3′UTR*, respective sgRNA plasmids, a respective repair template plasmid (injected at 50 ng/µl each), pCFJ104 (5 ng/µl), pCFJ90 (2.5 ng/µl), and pMA122 (10 ng/µl). pCFJ104, pCFJ90, and pMA122 were kind gifts from Erik Jorgensen (Addgene plasmids #19328, #19327, and #34873, respectively. The Cas9 expression plasmid was a kind gift from John Calarco (Addgene plasmid #46168). Correctly modified worms were selected based on hygromycin resistance after heat shock.

### Brood size and lethality assay

Hermaphrodite L4 larvae were individually placed on NGM plates containing *E. coli*OP50 to lay eggs at 22°C and transferred onto a fresh plate every 24 h. Each day, progeny was counted until egg laying ceased. For the lethality assay, offspring were incubated at 22°C and the number of adult/L4 animals was scored 48 h after the mother was removed. Sterile and semi-sterile (< 25 eggs) mothers were excluded from the assay.

### Larval development assay

To assess the time of development, ∼800 synchronized wild-type and *lat-1* L1 larvae were placed on *E. coli*OP50-seeded NGM plates and incubated at 22°C. After 8, 16, 24, 36, and 48 h, the number of larvae in different developmental stages (L1, L2, L3, L4, and adult) was scored.

### Ovulation rate assay

Ovulation rates were determined in hermaphrodites 24 and 96 h post-L4, respectively. Hermaphrodites were placed separately on *E. coli* OP50-seeded NGM plates, and eggs and oocytes inside the uterus were counted. After 4 h at 22°C, eggs/oocytes on the plate and inside the uterus of the mother were counted. The ovulation rate per gonad was calculated as ([eggs + oocytes in and out of uterus after 4 h] − [initial eggs + oocytes inside uterus])/(2 × 4 h).

### MitoTracker Red staining

To assess sperm movement in vivo, sperm was labeled using MitoTracker Red (Kubagawa *et al*. 2006). L4 males were kept isolated overnight to ensure sperm accumulation. The following day, young adult males were stained with 10 µM MitoTracker Red CM-H2XRos (Thermo Fisher) in M9 for 2 h in the dark at room temperature and then left overnight to recover on plates seeded with *E. coli*OP50. The next day, 25 stained males and 25 young adult hermaphrodites, which were first anesthetized using 300 µM levamisole (AppliChem), were mated on a 5-µl spot of overnight grown *E. coli*OP50. Copulation was monitored. Successfully inseminated hermaphrodites (indicated by transfer of fluorescent sperm into the uterus) were transferred separately into the wells of a 72-well plate containing 1.5 mM levamisole. Thereafter, hermaphrodites were mounted on 2% agarose pads and microscopy was conducted 1 h postinsemination. The resulting stack images of MitoTracker Red-stained sperm in the hermaphrodite uterus were evaluated as average intensity Z-projections. Correct localization of sperm was defined as proximal of the first egg next to the spermatheca, including all sperm around this egg. The percentage of correctly localized sperm was calculated as (mean fluorescence intensity of correctly localized sperm/mean fluorescence intensity of all sperm inside uterus).

### Sperm activation assay

Sperm activation was studied as previously described (Hammerquist *et al*. 2021) with minor modifications. L4 male nematodes were placed on separate NGM plates with *E. coli*OP50 and kept isolated to avoid premature sperm activation. The following day, nematodes were dissected in Sperm Medium buffer containing BSA and treated with 200 ng/µL pronase (Sigma) for 15 min. Following stimulation, differential interference contrast (DIC) microscopy was conducted within 5 min and sperm was scored as activated (with full pseudopod) or not activated (spherical, lobed, and spiked). At least 100 spermatids were scored per replicate.

### SYTO staining

Adult hermaphrodites (24 h post-L4) were washed off NGM plates with NGM lacking phosphate buffer and incubated overnight in the dark at room temperature with 33 µM SYTO 12 green fluorescent nucleic acid stain (Thermo Fisher) in NGM and a small amount of *E. coli*OP50. To remove the stained bacteria, worms were washed 3 times with NGM and transferred to NGM plates with fresh *E. coli*OP50 for 1 h. Subsequently, stained worms were anesthetized with 300 µM levamisole (AppliChem) in M9 and mounted on 2% agarose pads for microscopy.

### CED-1::GFP imaging

Adult *bcIs39[lim-7p::ced-1::GFP; lin-15(+)]* and *bcIs39[lim-7p::ced-1::GFP; lin-15(+)]*; *lat-1(ok1465)* hermaphrodites were anesthetized with 300 µM levamisole (AppliChem) in M9 and mounted on 2% agarose pads for microscopy. CED-1::GFP was scored per gonad.

### Antibody and DAPI staining

Antibody and DAPI stainings were performed on extruded germlines. For this purpose, germlines of precisely synchronized hermaphrodites were dissected, fixed, and stained as previously described (Francis *et al*. 1995; Jones *et al*. 1996). Adult hermaphrodites (24 h post-L4) were transferred into 300 µM levamisole (AppliChem) in 0.1% Tween in PBS (PBST) for immobilization, and gonad arms were exposed by cutting off the heads/tails with a scalpel blade. Gonads were either fixed in 4% formaldehyde/PBST solution for 15 min (DAPI only) or 10 min (antibody staining). After washing with PBST, the specimens were postfixed in 100% ice-cold methanol at -20°C for 5 min (DAPI only).

To boost the expression signals of genome-edited *LNT::GFP* (strain *lat-1(apr1[lat-1(1-581)::GFP^hygR^])*), antibody staining was performed following permeabilization in PBST + 0.1% Triton X-100 for 30 min and blocking with PBST + 1% BSA for 1 h at room temperature. Samples were subsequently incubated in rabbit anti-GFP antibody (Invitrogen, G10362) (1:100 in PBST + 1% BSA) overnight at 4°C. Goat anti-rabbit StarRED (Aberrior, 1:500 in PBST + 1% BSA with 1 ng/µl DAPI) (for overview gonad and spermatheca stainings) or goat anti-rabbit Alexa 568 (Invitrogen, 1:1,000 in PBST + 1% BSA with 1 ng/µl DAPI) [for distal tip cell (DTC) and gonadal sheath cell stainings] were used as secondary antibodies for 1 h at room temperature. After three brief wash steps in PBST, the gonads were mounted on 2% agarose pads in Fluoromount-G (Thermo Fisher) for microscopy. To stain gonads exclusively with DAPI, formaldehyde/methanol-fixed gonads were incubated for 2 h at 4°C in the dark with 1 ng/µl DAPI. Gonads were washed 3 times with M9 and mounted as described above. Because of the faint mCherry expression of *lat-1(knu846 [lat-1 KO/KI mCherry intronic loxP::hygR::loxP])*, strains carrying this marker were not fixed and gonads were mounted in their native form for microscopy.

### Microscopy

All specimens were imaged using confocal imaging techniques. DIC and fluorescence imaging were performed using a Leica SP8 microscope and an Olympus FluoView FV1000 microscope. Z-stacks were taken with a spacing of 0.5–2 μm, depending on the specimen (2 μm for whole worms, 0.5–1 μm for germlines, and 0.5 μm for sperm). Microscopic images were evaluated using Fiji (Schindelin *et al*. 2012) and ImageJ (Schneider *et al*. 2012).

### Oocyte size and area measurements

To asses oocyte size, strains of different genotypes expressing *tnIs13[pie-1p::vab-1::GFP; unc-119(+)], ltIs44[pie-1p::mCherry::PH(PLC1delta1); unc-119(+)]* containing a cell membrane as well as a cytoplasmic marker were utilized. Worms were grown at 25°C to induce marker expression, anesthetized at young adult stage with 300 µM levamisole (AppliChem) in M9, and mounted on 2% agarose pads for microscopy. Stack images of oocytes were acquired with stack borders set on the last visible part of oocyte membrane, visualized by mCherry fluorescence. Every 2 adjacent images were used to reconstruct part of the oocyte modeled as a truncated pyramid with the volume *V* = 1/3 × (slice spacing) × [*A*1 + *A*2 + sqrt(*A*1 × *A*2)], with *A*1 and *A*2 being the area enclosed by the oocyte membrane in both respective images. The full oocyte volume was determined by summing the volumes of all truncated pyramids.

For determining oocyte area, oocytes of L4 + 1-day-old hermaphrodites were anesthetized using 300 µM levamisole (AppliChem) in M9 and mounted on 2% agarose pads. Images of the middle section of an oocyte were taken using a microscope with DIC, and the area was measured using Fiji (Schindelin *et al*. 2012).

### Generation of transgenes

To obtain the constructs used in this study, recombineering was conducted. Existing protocols (Dolphin and Hope 2006; Tursun *et al*. 2009) were modified as previously described (Langenhan *et al*. 2009; Prömel *et al*. 2012) to generate LAT-1 transgenes using cosmids, PCR-amplified targeting cassettes, and positive antibiotic selection. All transgenes are based on a construct comprising the genomic locus of *lat-1* containing *lat-1p::lat-1(1-581)::GFP* (pTL20) (Prömel *et al*. 2012). To replace the promoter with tissue-specific promoters, a recombineering targeting cassette consisting of two parts, a spectinomycin selection cassette and the promoter sequence, was generated. For generating CRISPR/Cas9-based integrations, a plasmid-based approach was used as described previously (Chen *et al*. 2013; Dickinson *et al*. 2013; Friedland *et al*. 2013). For primer sequences, see Supplementary Table 1 in Supplementary File 1.

### *lag-2p::lat-1(1-581)::GFP (lag-2p::LNT::GFP,* pJL13)

A 2-kb promoter region of *lag-2* was amplified with primers lat1_1088F/lat1_1089R from genomic DNA of a mixed population of N2 and ligated into vector pCR2.1 using the TOPO TA Cloning Kit (Thermo Fisher). From this vector, the promoter was again amplified with primers lat1_1090F/lat1_1091R with the forward primer introducing a *Pst*I site and the reverse primer containing an overhang with a homology to the vector pTL20. In parallel, the spectinomycin resistance gene was amplified from the Gateway cloning vector backbone pDON223 (Invitrogen) with primers lat1_1080F (introducing an overhang with a homology to pTL20) and lat1_1081R (containing a *Pst*I site). Both fragments were digested by *Pst*I and ligated together using a T4 DNA ligase. The ligated cassette was subsequently recombineered into pTL20 (*lat-1p::lat-1(1-581)::GFP*) replacing the *lat-1* promoter using electrocompetent *E. coli* SW105 cells with a heat-induced recombinase of the λ-Red recombinase system (Warming *et al*. 2005).

### *plc-1p::lat-1(1-581)::GFP (plc-1p::LNT::GFP,* pJL14)

The promoter region of *plc-1* (2 kb) was obtained by amplification from genomic DNA of a mixed N2 population using primers lat1_1084F/lat1_1085R. Following ligation into vector pCR2.1 using the TOPO TA Cloning Kit (Thermo Fisher), the promoter was amplified with primers lat1_1086F/lat1_1087R. While the forward primer introduced a *Pst*I site, the reverse primer contained an overhang with a homology to the vector pTL20. In parallel, the sequence of the spectinomycin resistance gene was amplified from the Gateway cloning vector backbone pDON223 (Invitrogen) with primers lat1_1080F (containing an overhang with a homology to pTL20) and lat1_1081R (introducing a *Pst*I site). Both fragments were digested by *Pst*I and ligated with a T4 DNA ligase, and the resulting fragment was recombineered into pTL20 (*lat-1p::lat-1(1-581)::GFP*) replacing the *lat-1* promoter using electrocompetent *E. coli* SW105 cells with heat-induced recombinase of the λ-Red recombinase system (Warming *et al*. 2005).

### *lim-7p::lat-1(1-581)::GFP (lim-7p::LNT::GFP,* pDM3*)*

The promoter region of *lim-7* (2 kb) was retrieved by PCR with primers lat1_1082F (containing a *Pst*I site) and lat1_1083R (harboring a homology sequence to pTL20) from vector pOH323, which was a kind gift from Oliver Hobert (Columbia University, New York, NY, USA) (Hall *et al*. 1999). In parallel, primers lat1_1080F (containing an overhang with homologies to pTL20) and lat1_1081R (introducing a *Pst*I site) were used to amplify the spectinomycin resistance gene from the Gateway cloning vector pDON223 (Invitrogen). Both PCR products were digested with *Pst*I (NEB) and ligated using a T4 DNA ligase. The resulting fragment was recombineered into pTL20 (*lat-1p::lat-1(1-581)::GFP*) replacing the *lat-1* promoter using electrocompetent *E. coli* SW105 cells with heat-induced recombinase of the λ-Red recombinase system (Warming *et al*. 2005).

### Repair template for *lat-1(1-581)::GFP^HygR^ (LNT::GFP,* pSP231*)*

A 1.5-kb fragment of *lat-1(1-581)* fused to GFP was amplified from pTL20 (*lat-1p::lat-1(1-581)::GFP*) (Langenhan *et al*. 2009) as 5′ flanking site using primers lat1_1896F/lat1_1897R. A fragment encoding the hygromycin resistance gene was amplified from plasmid IR98 (Radman *et al*. 2013) using primers lat1_1898F/lat1_1921R. 1.5 kb of the 3′ UTR and downstream sequence of *lat-1* was amplified from plasmid pTL2 (Langenhan *et al*. 2009) (encoding the *lat-1* genomic locus) using primers lat1_1900F/lat1_1901R. The fragments were inserted into IR98 by Gibson assembly (Gibson *et al*. 2009) using primers lat1_1902F/lat1_1903R. This was performed using NEBuilder HiFi assembly (New England Biolabs) according to manufacturer's instructions, followed by transformation into chemically competent *E. coli* DH5α.

### sgRNA plasmids

For CRISPR–Cas9 engineering of endogenous loci, sgRNA was cloned into vector pJJR50 as described by Waaijers *et al*. (2016). pJJR50 was a kind gift from Mike Boxem (Addgene plasmid #75026). gRNA was cloned into *Bbs*I-digested pJJR50 using annealed oligonucleotides 5′-ggactggctccagtggacga-3′/5′-tcactcatctttgagctcgg-3′ with primer pairs lat1_1904F/lat1_1905R and lat1_1906F, lat1_1907R, respectively. Ligation was performed using a T4 ligase (Thermo Fisher) according to the manufacturer's instruction, followed by transformation into chemically competent *E. coli* DH5α cells.

### RNA extraction and rapid amplification of 5′ cDNA-ends with PCR

Total RNA was extracted from a mixed population of wild-type hermaphrodites using TRI Reagent (Sigma) according to the manufacturer's protocol and transcribed into cDNA with the second-generation 5′/3′ RACE Kit (Roche) in combination with the Expand High Fidelity PCR System (Roche) as described in the supplier’s instructions using 2 different gene-specific primers (lat-1_1772R and lat-1_1772R). Before the PCR, the 3′ end of the cDNA was tailed with dATP and TdT. The cDNA mix was directly utilized as a template for the 5′ rapid amplification of cDNA-ends with PCR (RACE-PCR). Amplification was run with an Oligo-d(T) anchor primer and the gene-specific primers above, respectively. PCR products were separated via gel electrophoresis, cut out, purified with the Wizard SV Gel and PCR Clean-Up System (Promega), and cloned into the vector pCR2.1 using the TOPO TA Cloning Kit (Thermo Fisher) for further analysis. For primer sequences, see Supplementary Table 1 in Supplementary File 1.

### Transcript variant analyses

Publicly available RNA-seq datasets from adult wild-type hermaphrodite *C. elegans* var. Bristol N2 were downloaded via the Sequence Read Archive (Leinonen *et al*. 2011; Kodama *et al*. 2012), and paired-end reads with an average length of 101 bases were aligned to the WBcel235 genome (Howe *et al*. 2017) using STAR (Dobin and Gingeras 2015) The mapped reads were assembled into transcripts and quantified by StringTie (version v2.1.3b) (Pertea *et al*. 2015) using the genome annotation as initial set of transcript variants. Full-length transcripts and newly identified transcripts were inspected (Supplementary Table 2 in Supplementary File 1), and further analyses including visualization were conducted with the Integrated Genomics Viewer (Robinson *et al*. 2011). Accession numbers and mapping statistics are given in Supplementary Table 3 in Supplementary File 1.

### Statistical analysis

Numerical assay data were analyzed with GraphPad Prism version 9.2.0 (GraphPad Software). If not stated otherwise, the data are presented in box plots with 90% confidence interval. Statistical analyses were performed using a two-tailed Student’s *t*-test for comparing two groups and a one-way ANOVA in combination with a Bonferroni post hoc test for comparing multiple groups, respectively. *P* values of *P* ≤ 0.05 were considered statistically significant. All details are given in the respective figure legends.

## Results

### LAT-1 controls sperm guidance in hermaphrodites

**Fig. 1.**
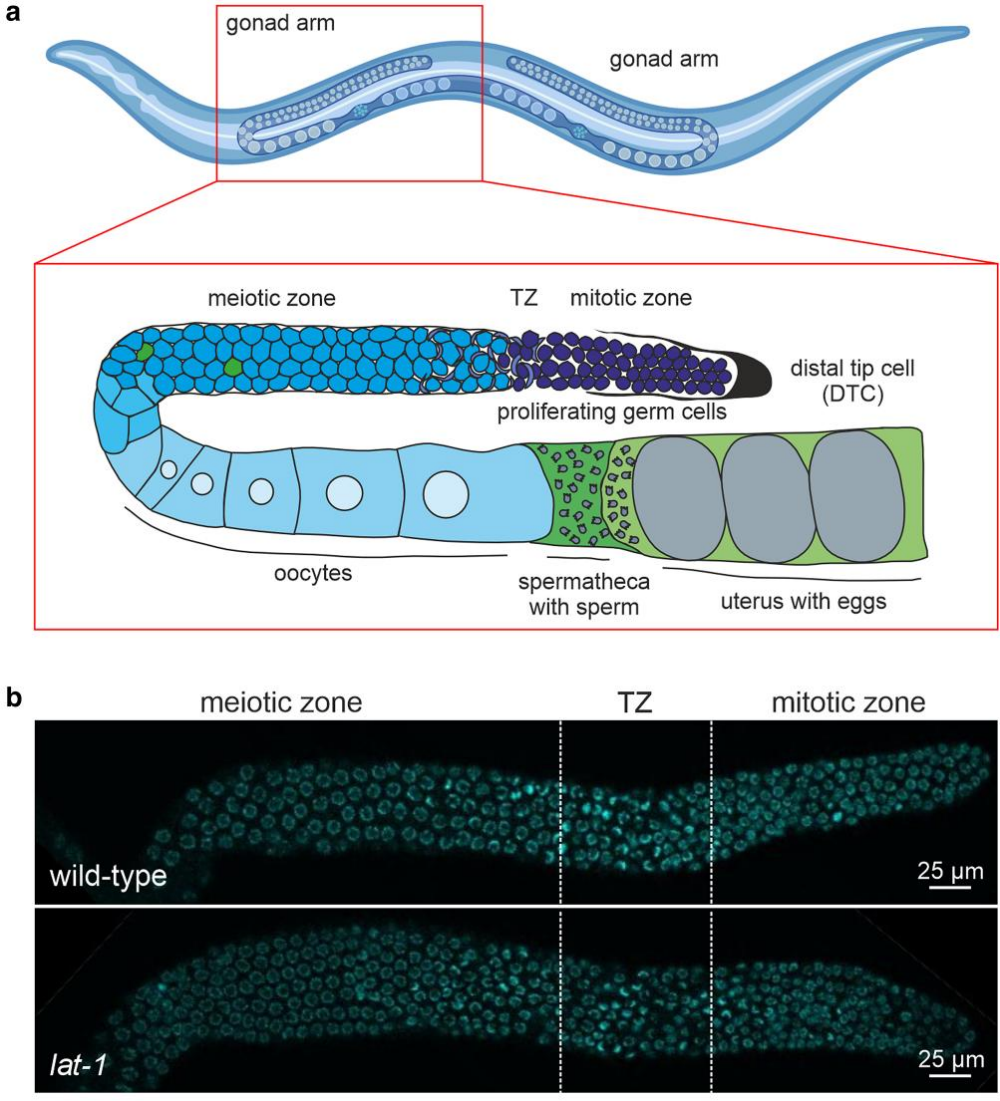
The *C. elegans* gonad. a) Shown is one of the two symmetrical U-shaped gonad arms of a *C. elegans* adult hermaphrodite. In the fourth larval stage (L4), a fixed number of sperm (∼150 per gonad) are produced and stored inside the spermatheca. Subsequently, the gonad switches to continuous oocyte production only. In the distal gonad, germ cell nuclei are not surrounded by a complete membrane but reside in a common cytoplasm. They continually self-renew by mitotic division in the proliferative (mitotic) zone, which is distally enclosed by the DTC. Subsequently, germ cells enter meiotic divisions (crescent-shaped nuclei) and progress further through meiosis, where some germ cells undergo apoptosis. Here, the cells are surrounded by the gonadal sheath cells (not shown). Near the loop, nuclei start to cellularize and to obtain individual plasma membranes. Due to coordinated contraction of the gonadal sheath cells, oocytes are pushed into the spermatheca, fertilized, and eggs are flushed into the uterus. After completing the first set of embryonic divisions, they are laid through the vulva (not shown). Parts of the figure were created in Biorender. Prömel, S. (2024) BioRender.com/u05l303. b) Dissected and DAPI-stained distal gonads of L4 + 1-day-old wild-type and *lat-1* hermaphrodites.

Previously, we showed that the LAT-1 N terminus (LNT) tethered to the membrane by its first transmembrane helix (aa 1-581, termed LNT) is sufficient to ameliorate the fertility defects in *C. elegans* homozygous for the null allele *lat-1(ok1465)* (hereafter referred to as *lat-1*), which are overall characterized by a reduced brood size (wild type: 228.2 ± 3.6; *lat-1*: 117.4 ± 4.0) (Langenhan *et al*. 2009; Prömel *et al*. 2012). These findings indicate that the unusual 7TM-independent/*trans* function of the receptor is involved in controlling aspects of reproduction of the self-fertilizing *C. elegans* hermaphrodite. The hermaphrodite gonad produces a fixed number of sperm during larval development, which are subsequently stored in the spermatheca, and variable amounts of oocytes are generated during adulthood (Fig. 1). We therefore asked whether the LAT-1 function affects only one or possibly several cellular processes or cell types culminating in the regulation of reproduction. To address this question, we dissected the defects of the *lat-1* mutants in greater detail.

**Fig. 2.**
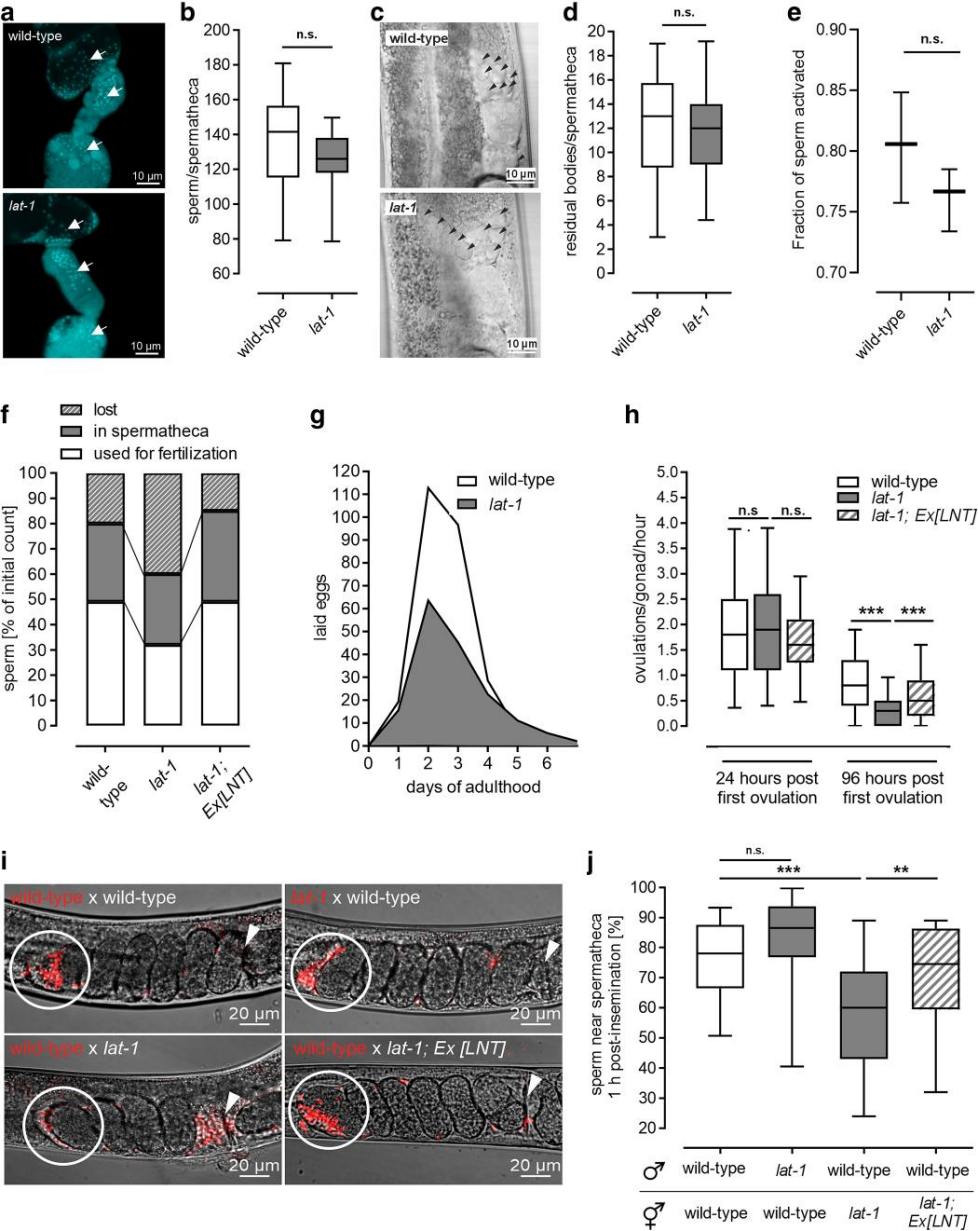
Nematodes lacking *lat-1* display impaired sperm movement. a) Spermathecae of L4 + 1-day-old *lat-1* mutant hermaphrodites contain the same amount of sperm (white arrows) as wild-type specimen. Gonads of adult hermaphrodites were dissected and subsequently stained with DAPI. b) Quantification of DAPI stainings as shown in (a) reveals that the number of sperm inside the spermatheca is indifferent from the one in wild-type nematodes. *n* ≥ 20 in 4 independent experiments. n.s., not significant. c) Residual bodies (black arrowheads) located close to the spermatheca in *lat-1* mutant and wild-type gonads. Shown are DIC images of unstained living L4 + 1-day-old hermaphrodites. d) Quantification of anucleate residual bodies based on DIC images as displayed in (c) revealed no significant difference in number between wild-type nematodes and *lat-1* mutants. Data from 4 independent experiments, *n* ≥ 24. n.s., not significant. e) Activation of spermatids by pronase treatment. Dissected spermatids from L4 + 1-day-old *lat-1* males showed a similar level of activation as wild-type controls. Data from 3 independent experiments, *n* ≥ 100. n.s., not significant. f) Sperm amount 48 h after the first ovulation. Sperm was DAPI-stained and sperm loss was calculated (for details, see ‘*Materials and methods*’). This loss is ameliorated in transgenic lines expressing LNT. It should be noted that due to experimental limitations, the initial sperm count in the spermatheca had to be taken from different animals than the count after 48 h. *n* ≥ 15. g) Hermaphrodites lacking *lat-1* exhibit a continuously smaller brood size over their entire reproductive period with the maximal reduction being visible on the second and third day of egg laying. *n* ≥ 75 in nine independent replicates. h) The ovulation rate in *lat-1* mutants is similar to the one in wild types 24 h after the first ovulation but decreases faster than in wild-type individuals or in *lat-1* mutants expressing the LNT. At 96 h after the first ovulation, it is significantly lower. Data of 4 independent experiments, *n* ≥ 51. n.s., not significant; ***P < 0.001. i) Representative images showing the distribution of male *lat-1* and wild-type sperm, respectively, after mating to hermaphrodites of different genotypes. The motility of *lat-1* sperm after mating with wild-type hermaphrodites appears to be intact, as indicated by correct localization near the spermatheca (white circle) 1 h postinsemination. Conversely, wild-type sperm inside *lat-1* hermaphrodites do not reliably localize next to the spermatheca and remain close to the vulva (white arrowhead). Mating assays were performed with wild-type and *lat-1* mutant nematodes in different constellations. Sperm was stained with MitoTracker Red by incubation of living young adult males prior to the mating, and sperm location was monitored 1 h after mating occurred. j) Sperm of wild-type males do not properly migrate to the spermatheca in *lat-1* mutant hermaphrodites after mating, while sperm of *lat-1* mutant males show no impaired movement toward the spermatheca of wild-type hermaphrodites. The former effect can be rescued by transgenic complementation of the LNT. Quantification was performed from images as shown in (h). Data were acquired in at least three independent experiments with *n* > 20 biological replicates. n.s., not significant; **P < 0.01; ***P < 0.001.

We first analyzed *lat-1* mutant sperm as previously obtained data led to the hypothesis that LAT-1 plays some role in sperm function (Prömel *et al*. 2012). Mutant hermaphrodites produced approximately the same amount of sperm as wild-type individuals (Fig. 2a and b), and the number of anucleate residual bodies generated during sperm production appeared to be constant (Fig. 2c and d). During larval stages, the germ cells that enter meiosis I proximally of the transition zone are called primary spermatocytes. Cytokinesis of meiosis I can be incomplete, i.e. the cytoplasm of the resulting secondary spermatocytes is still connected. During meiosis II, the secondary spermatocytes divide into spermatids and bud off the common cytoplasm to form individual haploid cells. The remaining anucleate cytoplasm is called a residual body and is eventually resorbed. Spermatids are activated to functional spermatozoa just before fertilization in the spermatheca (summarized in L'Hernault 2006). Our data indicate largely intact sperm development and the correct timing of the sperm–oocyte switch during the transition of the last larval stage into adulthood.

When crossing *lat-1* males with wild-type feminized hermaphrodites, we previously observed a reduced number of progeny compared to wild-type males (Prömel *et al*. 2012), suggesting either a reduced sperm function or decreased sperm transfer. As *lat-1* males also display a defective mating behavior (Matus *et al*. 2022), the observed reduced progeny of *lat-1* males could be attributed to sperm transfer during mating rather than sperm function. To exclude a defect in functionality of *lat-1* sperm, we treated spermatids from unmated males with pronase, which should activate them. As no difference was observed between wild-type and *lat-1* spermatid activation (Fig. 2e), we hypothesized that *lat-1* sperm was grossly functional.

As *lat-1* nematodes lay overall fewer fertilized eggs (Prömel *et al*. 2012) and thus use less sperm than wild-type worms in a given time period, this should result in an accumulation of residual sperm over time. To assess a possible change in the amount of sperm during the fertilization period, we examined sperm in the spermatheca 48 h after the first ovulation (Fig. 2f) together with the course of egg laying (Fig. 2g). Sperm amounts in the spermatheca 48 h after the first ovulation were not elevated, but slightly, albeit not significantly reduced compared to the amount in wild types. At the same time, significantly less sperm was used for fertilization indicating a loss of sperm during adulthood. This sperm loss was evaluated by subtracting the number of laid eggs and residual sperm within the spermatheca from the initial sperm count (Fig. 2f). As sperm loss can be caused by impaired motility or defective directed locomotion of sperm, which leads to them being flushed out during egg laying (summarized in Han *et al*. 2010), our data suggest that LAT-1 could play a role in sperm movement. Consistent with this hypothesis, the ovulation rate in *lat-1* mutants, which was initially similar to the one of wild-type worms, declined more rapidly over time (Fig. 2h). This is plausible as in *C. elegans*, ovulation is stimulated by signals produced in spermatozoa (reviewed in Han *et al*. 2010). Both phenotypes were rescued by the transgenic LNT (Fig. 2f and h), showing that the role in sperm movement is mediated by the 7TM-independent function of LAT-1. It should be noted that worms were precisely staged in all experiments, also to account for the fact that some *lat-1* mutants are developmentally slower than wild-type controls (Langenhan *et al*. 2009) (Supplementary Fig. 1 in Supplementary File 1).

To further assess the hypothesis that LAT-1 is essential for sperm movement, we monitored MitoTracker Red-labelled sperm after mating of males and hermaphrodites of different genotypes. Sperm of *lat-1* mutant males did not show impaired movement toward the spermatheca of wild-type hermaphrodites, indicating largely intact sperm motility (Fig. 2i and j). In contrast, wild-type sperm inside *lat-1* hermaphrodites did not localize near the spermatheca to the same extent as in the respective wild-type control. This defect was ameliorated by transgenic expression of the *LNT* in the hermaphrodite. Thus, our data indicate a defect in sperm guidance originating from *lat-1* hermaphrodites rather than impaired sperm function.

### LAT-1 modulates germ cell apoptosis and affects oocyte quality

**Fig. 3.**
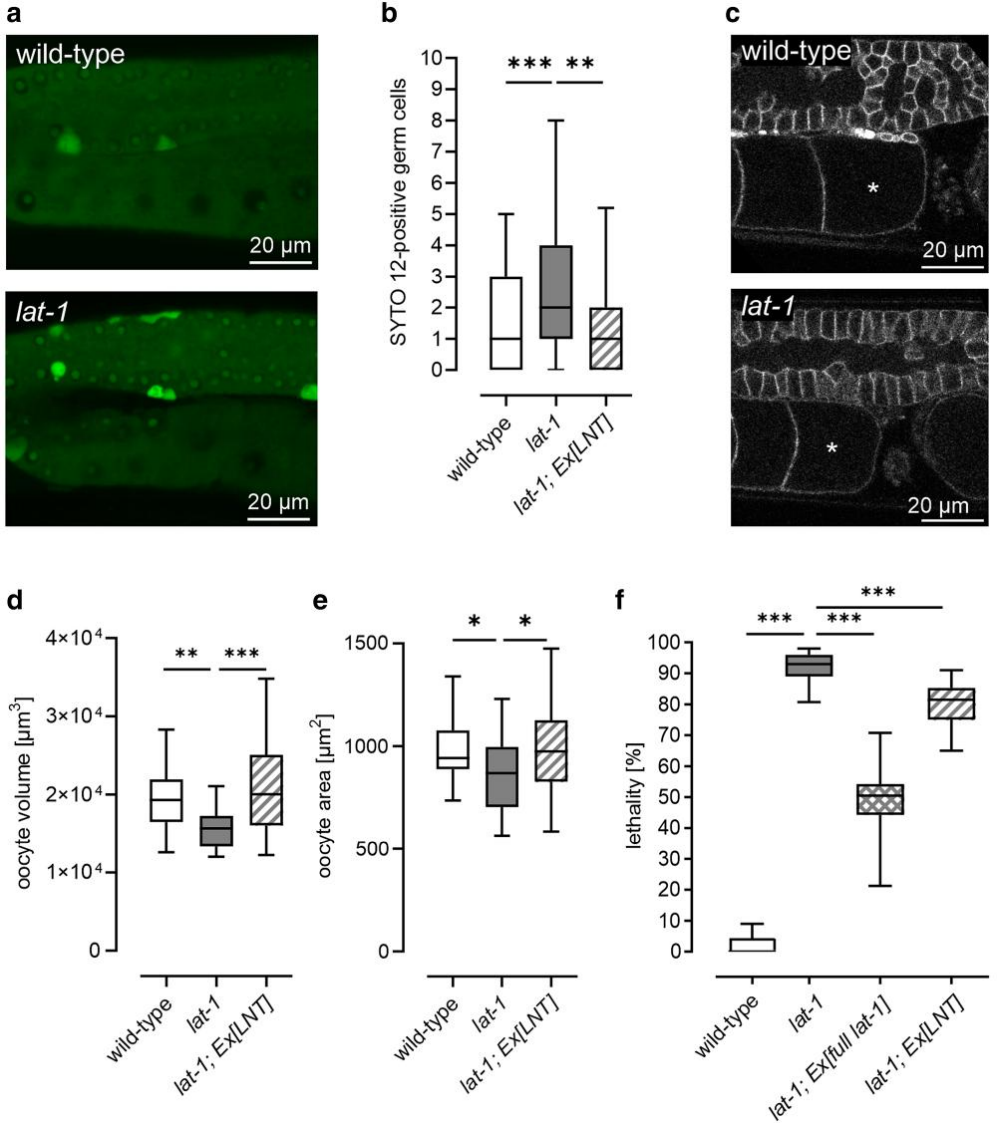
In the absence of LAT-1 function, germ cell apoptosis is increased. a) Germlines of *lat-1* mutants exhibit more SYTO 12-stained germ cells compared with wild-type controls. Living adult nematodes 24 h post-L4 were stained with SYTO 12 as a marker for apoptotic cells. Shown are representative images. b) Quantification of SYTO 12 stainings from (a). Reintroduction of the LNT rescues the increased number of apoptotic cells. Given are numbers per gonad. *n* ≥ 33. **P < 0.01; ***P < 0.001. c) Oocytes (marked with an asterisk) are significantly smaller in *lat-1* mutants than in wild-type individuals. Oocyte membranes were visualized in living young adult hermaphrodites by expression of the construct *pie-1p::mCherry::PH(PLC1delta1)*. Shown are representative images. d) Quantification of (c) reveals that oocyte size is decreased in *lat-1* mutants. This defect can be ameliorated by expression of the *LNT*. *n* ≥ 18. **P < 0.01; ***P < 0.001. e) Oocyte size determination by area measurements confirms data from (d). This way of measuring oocyte dimension was employed to supplement the volume measurements. Data from at least 5 independent experiments of area measurements, *n* ≥ 39, *P < 0.05. f) The LNT rescues embryonic lethality of *lat-1* mutants to a small but significant extent, while amelioration by the full-length *lat-1* construct is much stronger and is considered the maximum rescue that can be reached. Assays were performed in 4 independent experiments, *n* ≥ 30, ***P < 0.001.

Besides the effect on sperm movement, we found that the 7TM-independent mode of LAT-1 also affects oocytes. We observed an increase of germ cell apoptosis by SYTO 12-staining of *lat-1* mutant hermaphrodites, which was rescued by transgenic expression of the *LNT* (Fig. 3a and b). We also confirmed the increase in apoptotic bodies independently using a CED-1::GFP reporter (Zhou *et al*. 2001), which labels germ cells that are being engulfed by the somatic gonadal sheath cells (Supplementary Fig. 2 in Supplementary File 1). During adult stages, germ cells normally form oocytes, and it is a physiological process during *C. elegans* oogenesis that some of them undergo apoptosis (Fig. 1) (summarized in Gartner *et al*. 2008). While its role is debated, one possible function is to provide nutrients for other developing oocytes (Gumienny *et al*. 1999; Andux and Ellis 2008). Mutants of the apoptosis pathway (*ced-3* and *ced-4*) showed a reduced oocyte size (Andux and Ellis 2008). Thus, oocytes gaining too little nutrients are of inferior quality, reflected by reduced oocyte size (Andux and Ellis 2008) and increased embryonic lethality (Carranza-Garcia and Navarro 2019). Surprisingly, *lat-1* oocytes displayed both characteristics: smaller oocytes which can be ameliorated by the presence of the LNT (Fig. 3c–e), and increased lethality (Fig. 3f). As nematodes null for *lat-1* generally show embryonic lethality due to the receptor’s function in early development (Langenhan *et al*. 2009; Müller *et al*. 2015), it is impossible to assess oocyte quality by directly quantifying embryonic lethality. Thus, we investigated *lat-1* worms expressing the *LNT*. As this construct has been observed to rescue fertility defects, but not embryonic defects (Prömel *et al*. 2012), any amelioration of embryonic death detected in the corresponding strain is more likely to stem from maternal, postembryonic LAT-1 function. Comparison with *lat-1* mutants revealed that embryonic lethality is less pronounced in the presence of the LNT (Fig. 3f), indicating a cause other than developmental defects such as inferior oocyte quality. It must be noted that rescue by the LNT occurs to a much lesser extent than rescue by the full-length receptor, which is due to the fact that the latter one is essential for embryonic development (Langenhan *et al*. 2009; Müller *et al*. 2015).

These data point toward *lat-1* oocytes gaining too few nutrients, which can imply insufficient apoptotic events/clearance of apoptotic cells rather than too many. Therefore, *lat-1* worms may not display a genuine increase in apoptosis but have other defects resulting in apoptotic cell accumulation.

### LAT-1 elicits its functions from the somatic gonad in *trans*

**Fig. 4.**
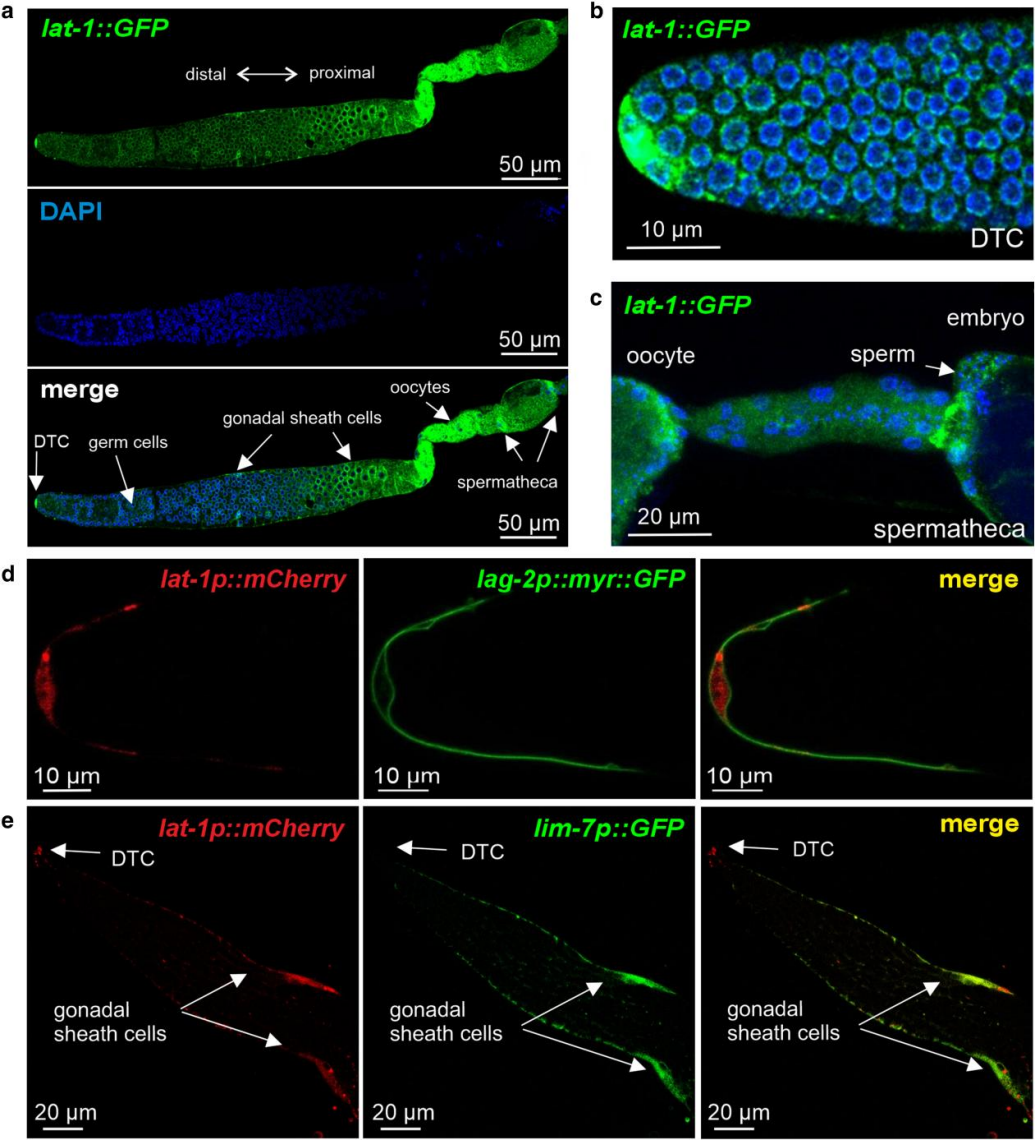
Expression of *lat-1* in the hermaphrodite gonad. a) The receptor is present in all somatic cells of the gonad as well as germ cells. b), d) *lat-1* is expressed in the DTC. It is further localized c) in the spermatheca and e) in gonadal sheath cells. For visualizing *lat-1* expression, either a CRISPR/Cas9 genome-edited single-copy integrated *LNT::GFP* (*lat-1(1-581)::GFP*) was used with signal amplification by an anti-GFP antibody (a–c) (for details, see ‘*Materials and methods*’) or a CRISPR/Cas9 genome-edited single-copy integrated *lat-1p::mCherry* (d, e) was employed. The DTC was visualized (d) by expressing *lag-2p::myr::GFP* and the gonadal sheath cells by using *lim-7p::GFP* (e). It should be noted that LAT-1 is not visible in germ cells in (d and e) likely due to the weak expression and the fact that no signal amplification by antibodies was employed here.

All functions of LAT-1 identified in this study, in sperm guidance, ovulation, and germ cell apoptosis/oocyte quality, have been shown to be 7TM-independent functions of the aGPCR. To gain insights into the mechanisms underlying these functions, we addressed the question, from which cells the LAT-1 function originates. Thus, we first generated a detailed expression profile of the receptor in the hermaphrodite gonad. Utilizing a CRISPR/Cas9 genome-edited *LNT::GFP (lat-1(1-581)::GFP)*, we observed *lat-1* expression in the entire somatic gonad of adult hermaphrodites, including the DTC, the gonadal sheath cells, and the spermatheca (Fig. 4a–c). Furthermore, LAT-1 seemed to be present in germ cells and developing oocytes. To verify expression in DTC and gonadal sheath cells, a CRISPR/Cas genome-edited transcriptional *lat-1p::mCherry* was combined with a marker for the DTC (*lag-2::myr::GFP*) (Fig. 4d) or the gonadal sheath cells (*lim-7p::GFP*) (Fig. 4e), respectively.

**Fig. 5.**
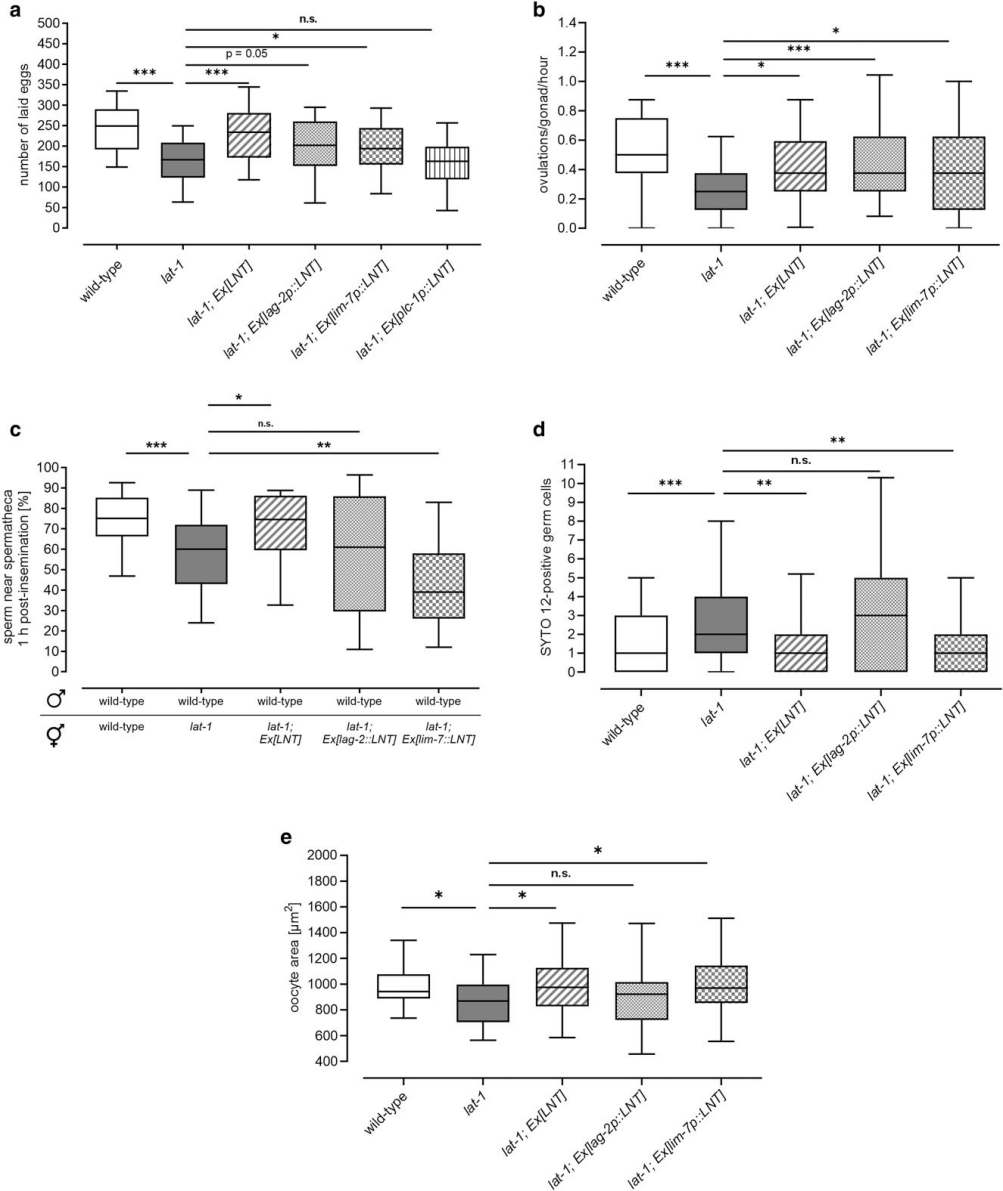
The LNT acts from somatic cells of the *C. elegans* gonad in a noncell autonomous manner. Tissue-specific expression of the LNT tethered to the membrane (LNT) using promoters with activity restricted to the DTC (*lag-2p::LNT*), the gonadal sheath cells (*lim-7p::LNT*), or the spermatheca (*plc-1p::LNT*) reveals whether a *trans* mode of the receptor fulfills its functions in different contexts on germ cells and in which location it is required. a) While expression of LNT in the DTC and the gonadal sheath cells generally results in a partial amelioration of the overall brood size defect of *lat-1* mutants, sole expression in the spermatheca (*plc-1p::LNT*) does not. b) The ovulation defect 96 h after the first ovulation of worms lacking *lat-1* is rescued by the LNT in both, the DTC and the gonadal sheath cells. c) Sperm migration to the spermatheca is rescued by the sole presence of LAT-1 neither in the DTC nor in the gonadal sheath cells. Data were acquired in at least three independent experiments with *n* > 20 biological replicates. n.s., not significant; *P < 0.05; **P < 0.01; ***P < 0.001. Note that some controls in this dataset (wild type, *lat-1*, *lat-1*; *Ex[LNT]*) are the same as in Fig. 2j. d) The increased apoptosis rate in *lat-1* mutants is ameliorated by the presence of the receptor in the gonadal sheath cells, but not in the DTC. Numbers are given per gonad. Data from at least 3 independent experiments, *n* ≥ 28. n.s., not significant; *P < 0.05; **P < 0.01; ***P < 0.001. Note that the controls in these data (wild type, *lat-1*, *lat-1; Ex[LNT]*) are the same as in Fig. 3b. e) The decreased oocyte size observed in *lat-1* mutant nematodes can be ameliorated by expressing the LNT in the gonadal sheath cells, but not in the DTC. Data from at least 5 independent experiments of area measurements, *n* ≥ 39. n.s., not significant; *P < 0.05. Note that the controls in these data (wild type, *lat-1*, *lat-1; Ex[LNT]*) are the same as in Fig. 3e.

To test whether the somatic gonad is the origin of the LAT-1 7TM-independent function, we specifically expressed the *LNT* in the DTC (*lag-2p::LNT*), the gonadal sheath cells (*lim-7p::LNT*), and the spermatheca (*plc-1p::LNT*) of *lat-1* mutants, respectively. First, brood size was quantified to assess whether the LNT in distinct cells has the capability to ameliorate the fertility defects in general (Fig. 5a). While expression in the DTC and gonadal sheath cells rescued at least partially (to a lesser extent than the LNT driven by its endogenous promoter), expression in the spermatheca did not. Focusing on the two tissues, in which *LNT* expression had an effect, we next elucidated whether it exerts a function from the DTC and/or the gonadal sheath cells specifically in the observed phenotypes: ovulation rate at 96 h after the first ovulation, sperm guidance, apoptosis, and oocyte size. While its presence in either the DTC or the gonadal sheath cells led to a partial amelioration of the ovulation rate (Fig. 5b), no significant rescue was achieved for sperm guidance (Fig. 5c). Receptor expression on the gonadal sheath cells had a rescuing effect on the increased apoptosis rate of germ cells in *lat-1* mutant hermaphrodites (Fig. 5d). The same rescuing effect was observed with regards to oocyte size (Fig. 5e).

These data highlight the fact that the LAT-1 7TM-independent/*trans* function is sufficient to control aspects of reproduction at least partially in a noncell autonomous manner.

### The LAT-1 transcript variant repertoire contains N terminus-only receptor versions

**Fig. 6.**
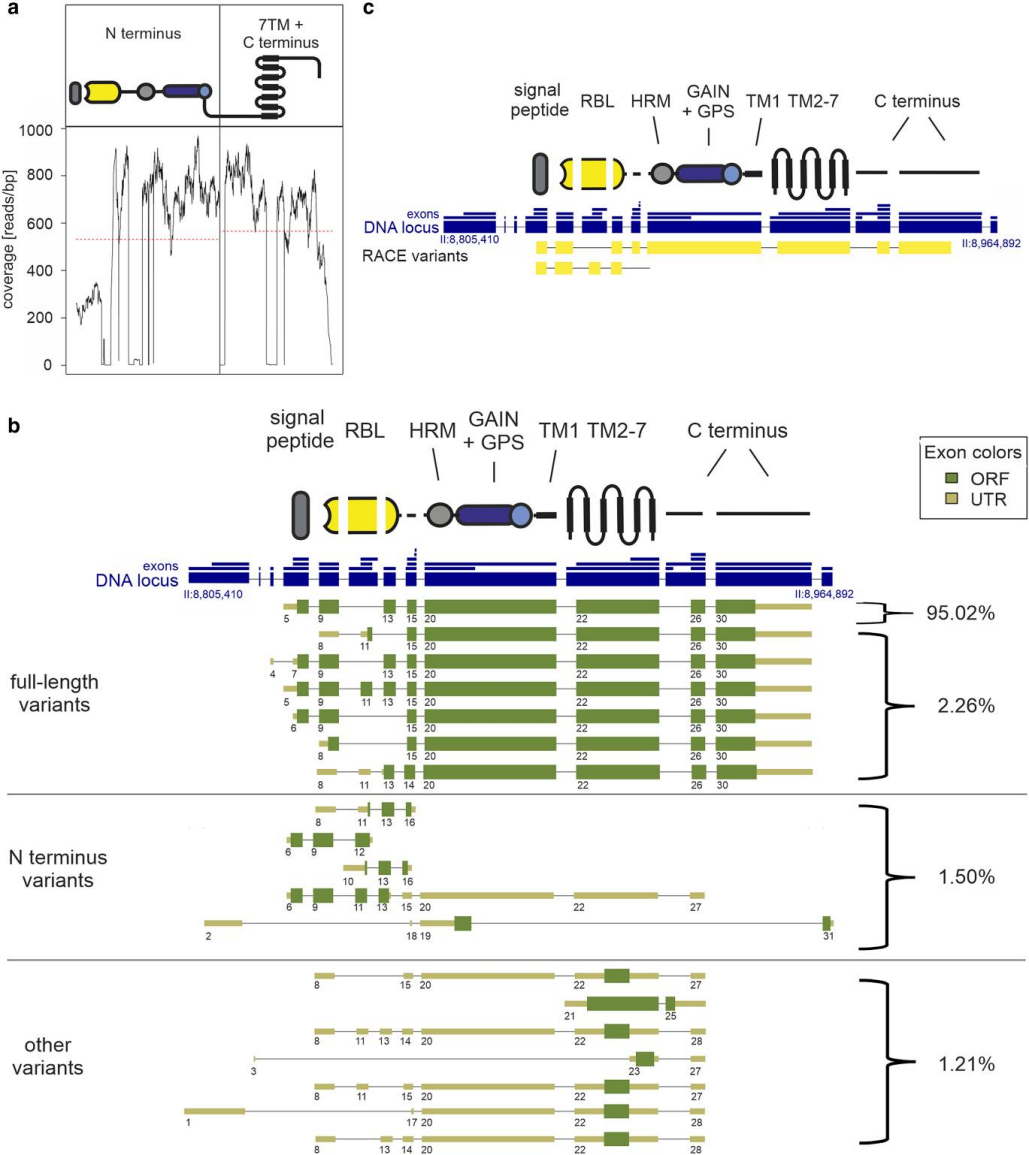
Several transcripts containing only the N terminus of LAT-1 exist. a) Read coverage of the *lat-1* locus extracted from available RNA-seq data of day 1 adult wild-type hermaphrodites (Chen *et al*. 2015) shows an almost uniform distribution of reads over the entire locus. b) Repertoire of *lat-1* transcript variants identified from RNA-Seq data (Chen *et al*. 2015). Relative abundance of each transcript variants was derived from Fragments Per Kilobase of transcript per Million mapped reads values estimated by StringTie (Supplementary Table 2 in Supplementary File 1). Several full-length variants exist which mostly differ in their N-terminal composition. Variants containing only the N terminus seem mostly not to be membrane-anchored. Other variants, which lack the N terminus, constitute about 1%. Only full-length variants with an incidence of more than 0.01% are depicted. The genomic locus of LAT-1 is shown with its longest exons (large boxes) and size-condensed introns (faint lines). All exons found in the analysis are separately plotted above the locus (small blue boxes). The individual exon arrangements of transcripts are shown numbered. Transcripts were defined as a numeric sequence of exons. The longest bona fide ORF is depicted in thick boxes, while the nonprotein coding 5′ and 3′ UTRs are displayed thinner and lighter. 3′ end exons with minor differences in length but identical 5′ splice acceptor sites are considered as one 3′ end exon. Different compositions of the 5′ start exon, 3′ end exon, and/or exons are considered as individual variants. The exact positions of the exons forming the variants are given in Supplementary Table 4 in Supplementary File 1. c) 5′ RACE analyses of wild-type hermaphrodites rendered among others the full-length variant, which has been shown to be the most abundant one in RNA-seq analyses. Further, a variant comprising the N terminus including RBL and HRM domain, but not the GAIN domain was amplified. RBL, rhamnose-binding lectin domain; HRM, hormone-binding domain; GPS, GPCR proteolytic site; GAIN, GPCR autoproteolysis-inducing domain.

As the LAT-1 7TM-independent functions act in a completely different biological context than that of its 7TM-dependent signal in embryogenesis (Langenhan *et al*. 2009; Prömel *et al*. 2012), the question arose as to why such a large receptor molecule is produced, when only the extracellular entity is mediating a function in distinct contexts. The release of the N terminus by cleavage of the aGPCR at the GPS, the motif in the GAIN domain capable of autoproteolysis, could be one mechanism. However, our previous findings indicate that cleavage is not essential for overall LAT-1 function (Prömel *et al*. 2012). Interestingly, a recent study suggests the presence of several premature poly-A sites within *lat-1* transcripts that, among others, render LAT-1 molecules consisting only of the N terminus (Tourasse *et al*. 2017). To gain insights into the prevalence of such variants, we analyzed existing transcriptome data generated by RNA-Seq from day 1 adult wild-type hermaphrodites (Chen *et al*. 2015), in which the coverage was sufficient for splice variant analyses (Fig. 6a). Generally, with more than 20 transcript forms, the repertoire of *lat-1* variants can be considered extensive. Although the largest fraction consisted of the entire receptor molecule, ∼1.5% of the variants comprised only the N-terminal exons in different combinations and versions, which are potentially able to transmit the 7TM-independent/*trans* function (Fig. 6b). A RACE-PCR from the mRNA of wild-type hermaphrodites confirmed the general presence of N terminus-only variants (Fig. 6c, Supplementary Fig. 3 in Supplementary File 1), suggesting that these variants of *lat-1* exist in vivo. It has to be noted that, to ensure correct transcript composition, rescue analyses were always performed with constructs containing the genomic locus of *lat-1* or modifications of it.

## Discussion

It is becoming increasingly clear that the highly unusual dual mode of classical *cis* signaling via G proteins and *trans* functions mediated solely through the N terminus (independent of 7TM and C terminus) are inherent to several aGPCR (Langenhan *et al*. 2009; Prömel *et al*. 2012; Patra *et al*. 2013; Tu *et al*. 2018; Ward *et al*. 2018). While the *cis* mode is relatively well studied in many cases, knowledge on the molecular details as well as the impact of the *trans* function is sparse, limiting our understanding of how such functions are integrated in multicellular settings. In the present study, we show that the 7TM-independent/*trans* mode is a frequently employed concept involved in different physiological and cellular contexts and that, in contrast to other aGPCR (Tu *et al*. 2018; Ward *et al*. 2018), the latrophilin homolog LAT-1 does not seem to engage in simultaneous bidirectional signaling (*cis* and *trans*) in these contexts. We identified LAT-1 N-terminal activity involved in different aspects of the *C. elegans* reproductive system, jointly balancing the brood size of the nematode. Thereby, the 7TM-independent/*trans* mode of LAT-1 action affects both gamete types and their function with seemingly diverse effects on sperm movement, ovulation, and germ cell apoptosis as well as quality.

Although it does not appear to be involved in sperm development or activation, the LAT-1*trans* function seems to be essential for sperm locomotion. The corresponding defect caused by the absence of the receptor is characterized by sperm loss occurring in the course of *lat-1* mutant adulthood (Fig. 2), due to impaired sperm movement. Possible reasons for this can generally be faulty sperm guidance cues or defective sperm. Our analyses strongly suggest LAT-1 to be involved in the sperm guidance signaling machinery of the hermaphrodite rather than sperm function per se, thereby affecting sperm movement. Many species use chemo-attractants to guide sperm to oocytes to ensure efficient fertilization. The anatomy of the *C. elegans* reproductive tract stresses the need for such signals, because as oocytes are pushed through the spermatheca and the uterus, sperm are dragged along and could be lost to the environment. Thus, a mechanism to lead them back to the spermatheca and localize them to the next oocyte is essential. Similarly, male sperm need to be able to localize the hermaphrodite's oocytes after being transferred into the uterus (summarized in Han *et al*. 2010). The likely origin of a chemo-attractant in *C. elegans* is the oocytes themselves. However, the spermatheca might also play a role in the establishment of the attractive signal (Kubagawa *et al*. 2006). The most likely candidates for chemo-attractants are F-series prostaglandins that are synthetized from polyunsaturated fatty acids (PUFA), as defects in genes involved in the metabolism of PUFA in the intestine (*fat-1-4*) and their uptake into the oocytes (*rme-2*, *daf-1*) lead to impaired sperm guidance (Kubagawa *et al*. 2006; Hoang *et al*. 2013; Hu *et al*. 2021). Other enzymes (*cyp-31A2*, *cyp-31A3*, and *emb-8*) are thought to downregulate prostaglandin synthesis within eggs in the uterus to uphold the signaling gradient toward the unfertilized oocytes (Hoang *et al*. 2013). Future studies will need to shed light on the question where LAT-1 can be placed in the mechanisms. We hypothesized that the reduced ovulation rate with time is related to the sperm movement defect as it correlates with the observed sperm loss. It is well established that ovulation is coordinated by sperm together with oocytes and gonadal sheath cells (Clandinin *et al*. 1998; Bui and Sternberg 2002; Han *et al*. 2010). However, our data suggest that it is likely that LAT-1 has an additional role in ovulation especially as the defects in ovulation can at least partially be rescued by expression of *lat-1* in the DTC or the gonadal sheath cells, while the sperm guidance defects cannot. It might also be conceivable that the guidance signal by transgenic overexpression is not strong enough to guide the sperm all the way back to the spermatheca but might be strong enough to keep them in the uterus, where they can contribute to signals stimulating ovulation.

We also found increased numbers of apoptotic cells in the proximal part of *lat-1* mutant germlines indicating a role of the receptor in programmed cell death (Fig. 3). Future analyses will need to clarify whether this is due to an increased rate of apoptosis itself or defective clearance of apoptotic germ cell corpses. However, the reduced oocyte size and increased embryonic lethality that can be attributed to the 7TM-independent/*trans* mode of LAT-1 hint toward reduced oocyte quality, which makes the latter hypothesis more likely. A function of LAT-1 in the regulation of apoptosis is intriguing since the mouse latrophilin LPHN1/ADGRL1 (Zuko *et al*. 2016), as well as some other aGPCR (Hsiao *et al*. 2015; Shiu *et al*. 2022), have previously been associated with programed cell death.

As these newly identified roles of LAT-1 are exerted solely by its 7TM-independent mode, but *lat-1* is expressed in germ cells as well as in the surrounding somatic cells, the question arose whether the receptor functions noncell autonomously and thus, in *trans*. Our analyses showed that the function in ovulation can be exerted by the LNT from both the DTC and the gonadal sheath cells, while in apoptosis and oocyte size, its presence is specifically required in the gonadal sheath cells rather than the DTC (Fig. 5). This suggests that the receptor indeed seems to fulfill its functions in these contexts noncell autonomously from the somatic germline. However, as the detected differences are small, which is, for instance, highlighted by the fact that no significant effect gradations in the rescue levels of the LNT from DTC and gonadal sheath cells were observed (Fig. 5a), an additional contribution from other tissues or the germ cells cannot be fully excluded. This could also apply to sperm locomotion (Fig. 5c), where no significant rescue by LAT-1 in any of the tested tissues was observed. Another possible explanation for this result is that the receptor might need to be present in all tissues simultaneously to effectively influence sperm guidance.

There is a plethora of physiological implications of LAT-1 identified in this study and previously (Prömel *et al*. 2012; Matus *et al*. 2022), and it is conceivable that several more LAT-1 functions exist. However, it remains to be determined which of these functions employ similar mechanisms on a molecular level. Despite the clear evidence that multiple aGPCR are able to engage in 7TM-independent/*trans* modes of action (Prömel *et al*. 2012; Patra *et al*. 2013; Patat *et al*. 2016; Tu *et al*. 2018; Ward *et al*. 2018), it is still debated whether these functions are used for intercellular signaling or if they can be sufficiently explained by a role in adhesion or even other modes of action. While adhesive components of LAT-1 cannot be excluded, the general morphology of *lat-1* mutant gonads seems intact (Fig. 1b) making a sole function as a structural component of cell–cell adhesion complexes less likely.

In the current study, we were not able to identify a clear canonical *cis* function complementing the *trans* function in *C. elegans* fertility (Fig. 5). This was a surprising finding since other aGPCR (Tu *et al*. 2018; Ward *et al*. 2018) seem to engage in simultaneous bidirectional *cis* and *trans* signaling from the same cells. In *C. elegans*, *cis* and *trans* functions seem to be separated both spatially and/or temporally, being involved in different cell types and developmental stages. It is known that the LAT-1*cis* function is essential for early embryonic development (Langenhan *et al*. 2009). Conversely, neuron morphogenesis in *C. elegans* was found to be mainly regulated by a *trans* function (Matus *et al*. 2022), with some evidence for *cis* functions that were not functionally essential. Similarly, in other postembryonic functions such as sperm guidance, ovulation, and germline apoptosis, *trans* functions seem to dominate. It should be noted that phenotypic analyses are never exhaustive and future analyses could unravel LAT-1*cis* effects in adult nematodes and *trans* functions in the embryo. Nevertheless, it would be intriguing to find out what molecular switch could cause such differences in the molecular mechanisms employed by LAT-1.

Intriguingly, there is some evidence that *trans* functions can be separated at a molecular level from the *cis* functions due to alternative splicing (Fig. 6). Thereby, isolated N-terminal variants can be expressed in vivo, and it is plausible that they are regulated separately from the full-length receptor. These data are also in concordance with recent studies on other aGPCR showing a huge tissue-specific transcriptional variability as a potential common principle in the regulation of aGPCR function (Boucard *et al*. 2013; Knierim *et al*. 2019; Röthe *et al*. 2019). It should be noted that no information is available on the presence of N-terminal variants selectively in the *C. elegans* somatic gonad or germ cells, only data on whole adult hermaphrodites are accessible. The small proportion of N-terminal variants (∼2%) might be due to this heterogeneity of the samples, i.e. the potentially small contribution of *trans* function-exerting cell types (such as the few cells of the somatic gonad) to the total mRNA pool. It remains to be determined whether these N-terminal variants are translated into protein and it is also conceivable that the receptor fulfills its 7TM-independent function as an intact full-length molecule. Alternatively, it cannot be excluded that the full-length aGPCR is produced but mediates the 7TM-independent function by the N terminus liberated through autoproteolysis at the GPS. This mechanism of self-cleavage, which is a hallmark feature of many aGPCR, has been described for LAT-1, but in previous studies we found that cleavage does not have a major impact on receptor function (Prömel *et al*. 2012).

In summary, we show that the LNT acts in *trans* in several, distinct physiological settings, underlining the importance as well as the versatility of this mode of action. We further demonstrate that next to the possibility of liberating the extracellular domains of aGPCR via autoproteolysis, alternative splicing can generate receptor N termini to exert 7TM-independent *trans* functions. It would be highly interesting to see whether the characteristics of *trans* signaling found for the prototypic aGPCR LAT-1 in this study are conserved among species and are moreover applicable to the entire class of aGPCR.

## Supplementary Material

jkae206_Supplementary_Data

## Data Availability

*C. elegans* strains and plasmids are available upon request. The authors affirm that all data necessary for confirming the conclusions of the article are present within the article, figures, and supplementary figures and tables. Supplemental material available at G3 online.
