# Supplementary material for: The N terminus-only (trans) function of the adhesion G protein-coupled receptor latrophilin-1 controls multiple processes in reproduction of Caenorhabditis elegans
Source: G3 (Bethesda). 2024 Sep 7;14(11):jkae206. doi: 10.1093/g3journal/jkae206 (PMC11540312; doi:10.1093/g3journal/jkae206)
Supplement: jkae206_Supplementary_Data [file jkae206_supplementary_data.pdf]

SUPPLEMENTAL INFORMATION

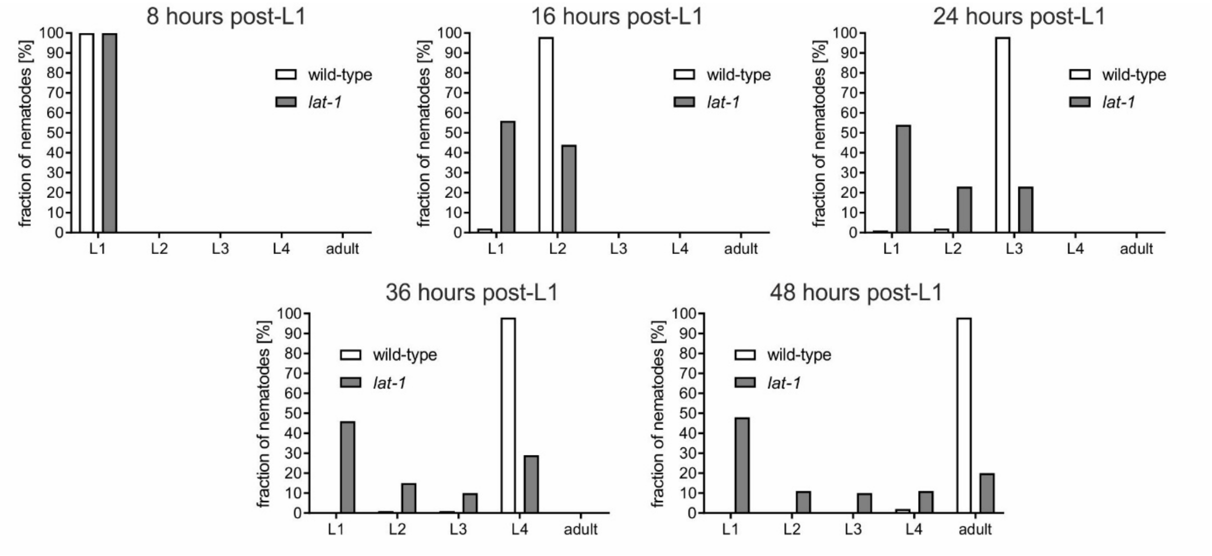

Figure S1. ***lat-1*** **nematodes display an impaired larval development.** A synchronized L1 population of nematodes was evaluated for progression through the larval stages up to adulthood at 8, 16, 24, 36, and 48 hours post-L1. While wild-type worms show a fast and synchronized progression, some *lat-1* nematodes arrest in early larval stages. However, a small percentage of *lat-1* mutants reach L4/adulthood at the same time as wild-type animals. Data from 4 independent experiments, n ≥ 700.

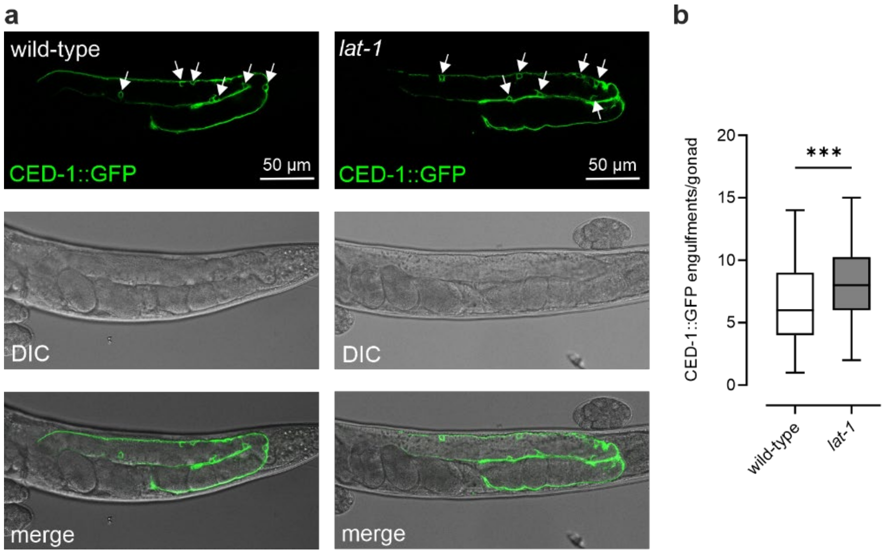

Figure S2. The number of engulfments visualized by CED-1::GFP is increased in ***lat-1*** **hermaphrodites.** (a) Representative images of CED-1::GFP in wild-type and *lat-1* hermaphrodites. (b) Quantification of GFP shown in (a) reveals that the number of engulfments is increased in *lat-1* hermaphrodites compared to wild-type nematodes. n ≥ 20 in 3 independent experiments. *** $p < 0.001$.

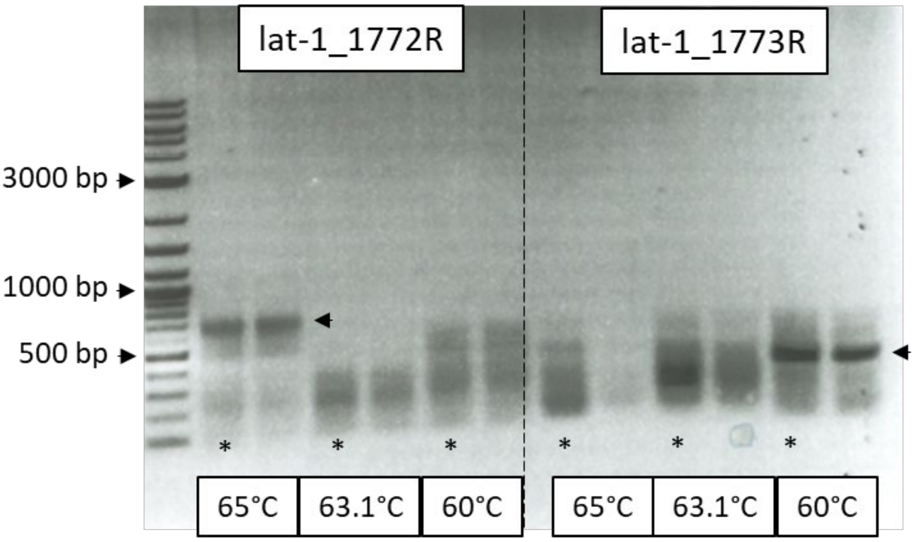

Figure S3. Exemplary gel picture of the 5’ rapid amplification of cDNA-ends with PCR (RACE-PCR). The RACE was performed with wild-type RNA with two different primers (lat-1_1772R binding in exon 5 and lat-1_1773R binding to the exon 4/exon 5 junction) at three different annealing temperatures (65 °C, 63.1 °C and 60 °C). Every sample was used both undiluted (*) and diluted (1:20 in water) within the PCR and then separated on a 2% agarose gel containing ethidium bromide gel. Band sizes for variants are 516 bp/505 bp (arrow left) and around 635 bp (arrow right) depending on the selected primers. Other visible bands did not yield any product.

Table S1. Sequences of primers used in the study.

Primer	Sequence 5'-3'
lat1_1080F	TTGTTTGTGAGTTTTCGTTTTCGCCTTTATCTTGTTATTTGAATCCGAATCCCTATAGTGAGTCGTATTACATGGTCATA
lat1_1081R	ATTGCTGCAGGATCTTTTCTACGGGGTCTGACGCTCAGTG
lat1_1082F	GATCCTGCAGCAATGCTTCCGAAAACCTCCGAGTTTTTCG
lat1_1083R	GCTACCAGAATCGTTTGGAGCAACGAATAAGTCGTTTTGTTACGTCGCATGCCGTTGAACAGATATAGAAGTTTGTGATT
lat1_1084F	CTTAACTGTCAGATTTCAAAAAAAAAAGGA
lat1_1085R	CTGTAATTGTCTCATATTTTGATTTTAAAA
lat1_1086F	GATCCTGCAGCTTAACTGTCAGATTTCAAAAAA
lat1_1087R	GCTACCAGAATCGTTTGGAGCAACGAATAAGTCGTTTTGTTACGTCGCATCTGTAATTGTCTCATATTTTGATTTTAAAAGAGTAAACAC
lat1_1088F	TTTGAATCCGAAAAAGTCTCAAAAAGCAAA
lat1_1089R	AAAAGGCAAATTTGAAAAGTGTTGTTGGCT
lat1_1090F	GATCCTGCAGTTTGAATCCGAAAAAGTCTCAAAAAGCAAA
lat1_1091R	GCTACCAGAATCGTTTGGAGCAACGAATAAGTCGTTTTGTTACGTCGCATAAAAGGCAAATTTGAAAAGTGTTGTTGGC
lat-1_1772R	AACGTCCGAATTGAGGGCAT
lat-1_1773R	TGAGGGCATCCTTTTGAGCC
lat1_1896F	CAGCGGATAACAATTTCACAtgcattttattttagcaatttaatgg
lat1_1897R	CTCCTTGCGTTCATCTTCATAACACCCCTTGTATTACTGT
lat1_1898F	ACAGTAATACAAGGGGTGTTATGAAGATGAACGCAAGGAG
lat1_1921R	acgcatcaaagatatggagaAGTTGAAACAGTTATGTTTGGT
lat1_1900F	ATTTAAATTTTCAGGAGTAAtctccatatctttgatgcgt
lat1_1901R	GGTTTTGGCGCGCCTTTAAAactgtcaaactttgagagattg
lat1_1902F	atctctcaaagtttgacagtTTTAAAGGCGCGCCAAAACC
lat1_1903R	aattgctaaaataaaatgcaTGTGAAATTGTTATCCGCTG
Oligo d(T)-Anchor Primer	GACCACGCGTATCGATGTCGACTTTTTTTTTTTTTTTTV<br>(V=A, C or G)
PCR Anchor Primer	GACCACGCGTATCGATGTCGAC

Table S2. Raw FPKM values derived from StringTie deduced from (CHEN *et al.* 2015).

	Transcripts		FPKM Sample1	% (of all *lat-1* transcripts)	FPKM Sample2	% (of all *lat-1* transcripts)	FPKM Sample3	% (of all *lat-1* transcripts)	% (mean)	
lat-1 transcripts	all transcripts in samples		46.29	100%	52.30	100%	44.4995	100%	100%	
Full-length complete	5,9,13,15,20,22,26,30	ADGRL1_wt_1.19 ADGF	43.75	94%	50.03	95.66%	42.23	94.90%	95.019%	**95.02%**
Full-length variants										
	4 7 9 13 15 20 22 26 30	ADGRL1_wt_1.15	0.836475	1.81%	0.00	0.00%	0.00	0.00%	0.602%	**2.29%**
	5 9 11 13 15 20 22 26 30	ADGRL1_wt_1.16_(mo)	0.459209	0.99%	0.00	0.00%	0.00	0.00%	0.331%	
	6 9 15 20 22 26 29	ADGRL1_wt_3.19_(mo)	0	0.00%	0.00	0.00%	0.38	0.84%	0.281%	
	8 11 13 14 20 22 26 29	ADGRL1_wt_3.12	0	0.00%	0.00	0.00%	0.09	0.20%	0.067%	
	8 11 15 20 22 26 30	ADGRL1_wt_1.28<br>ADGRL1_wt_3.17	0.399316	0.86%	0.00	0.00%	0.57	1.28%	0.713%	
	8 15 20 22 26 30	ADGRL1_wt_1.36	0.37441	0.81%	0.00	0.00%	0.00	0.00%	0.270%	
N terminus variants										
	10 13 16	ADGRL1_wt_3.35	0.0000	0.00%	0.0000	0.00%	0.2254	0.51%	0.253%	**1.50%**
	2 18 19 31	ADGRL1_wt_1.56	0.0615	0.13%	0.0000	0.00%	0.0000	0.00%	0.044%	
	6 9 11 13 15 20 22 27	ADGRL1_wt_2.2	0.0000	0.00%	0.2350	0.45%	0.0000	0.00%	0.225%	
	6 9 12	ADGRL1_wt_2.6	0.0000	0.00%	0.3024	0.58%	0.0000	0.00%	0.289%	
	8 11 13 16	ADGRL1_wt_2.5<br>ADGRL1_wt_3.34	0.0000	0.00%	0.6791	1.30%	0.0353	0.08%	0.689%	
other variants										
	8 11 15 20 22 27	ADGRL1_wt_2.3	0.000	0.00%	0.257	0.49%	0.000	0.00%	0.16%	**1.21%**
	8 13 14 20 22 28	ADGRL1_wt_3.24	0.000	0.00%	0.000	0.00%	0.023	0.05%	0.02%	
	8 15 20 22 27	ADGRL1_wt_2.4	0.000	0.00%	0.518	0.99%	0.000	0.00%	0.33%	
	21 25	ADGRL1_wt_3.36	0.000	0.00%	0.000	0.00%	0.231	0.52%	0.17%	
	3 23 27	ADGRL1_wt_2.7	0.000	0.00%	0.207	0.40%	0.000	0.00%	0.13%	
	1 17 20 22 28	ADGRL1_wt_3.28	0.000	0.00%	0.000	0.00%	0.116	0.26%	0.09%	
	8 11 13 14 20 22 28	ADGRL1_wt_3.18	0.000	0.00%	0.000	0.00%	0.408	0.92%	0.31%	

Table S3. Accession numbers and mapping statistics for transcript analysis of *lat-1* variants.

GEO Accession	SRA run	raw reads	uniquely aligned raw reads [%]
GSM1862268	SRR2185654	2 x 57999404	94.52
GSM1862269	SRR2185655	2 x 40607164	94.76
GSM1862270	SRR2185656	2 x 49090847	94.62

Table S4. Exon positions of *lat-1* variants analyzed in this study. Given are the identified exons for the *lat-1* gene (numbering refers to the reference *C. elegans* genome (WBcel235)).

Exon	Chromosome	Start	End
1	II	8805410	8805979
2	II	8805628	8805979
3	II	8846108	8846118
4	II	8890831	8890855
5	II	8896750	8896987
6	II	8896839	8896987
7	II	8896841	8896987
8	II	8899797	8899984
9	II	8899798	8899984
10	II	8903278	8903494
11	II	8903388	8903494
12	II	8903300	8903521
13	II	8903607	8903719
14	II	8903832	8903929
15	II	8903844	8903929
16	II	8903844	8903930
17	II	8903913	8903929
18	II	8903916	8903929
19	II	8904012	8904492
20	II	8904012	8905264
21	II	8905967	8906846
22	II	8906060	8906846
23	II	8906575	8906846
24	II	8906914	8906981
25	II	8906915	8907289
26	II	8907152	8907287
27	II	8907152	8907288
28	II	8907152	8907289
29	II	8907725	8908630
30	II	8907725	8908636
31	II	8964794	8964892

REFERENCES

Chen, A. T., C. Guo, O. A. Itani, B. G. Budaitis, T. W. Williams *et al.*, 2015 Longevity Genes Revealed by Integrative Analysis of Isoform-Specific daf-16/FoxO Mutants of Caenorhabditis elegans. Genetics 201**:** 613-629.
